# How to take action beyond ambulatory glucose profile: Latin American expert recommendations on CGM data interpretation

**DOI:** 10.1186/s13098-025-01702-y

**Published:** 2025-05-08

**Authors:** Luis Eduardo Calliari, Álvaro Contreras Sepúlveda, Nicolás Coronel-Restrepo, Laura Kabakian, Rodrigo N. Lamounier, Emma Picasso, Adrian Proietti, Alex Ramírez-Rincón, Alicia E. Yépez-Rodriguez

**Affiliations:** 1 Department of Pediatrics, Santa Casa School of Medical Sciences, São Paulo, Brasil; 2 Clínica Universidad de los Andes, Santiago, Chile; 3 Clínica Medellín, Grupo Quirón Salud, Medellín, Colombia; 4 Centro Médico Integral (CMI), Buenos Aires, Argentina; 5 Internal Medicine Department, School of Medicine, Federal University of Minas Gerais (UFMG), Belo Horizonte, Brazil; 6 Pediatric Endocrinology Department, Clínica EnDi y Corporativo Hospital Satélite (CHS), Ciudad de Mexico, México; 7 Endocrinology and Diabetes Department, Kynet Integral, Buenos Aires, Argentina; 8 Scientific Direction, IPS Especializada Diabetes SURA, Clínica Auna Las Américas, Universidad Pontificia Bolivariana, Medellín, Colombia; 9 Endocrinology Department, Corporativo Hospital Satélite (CHS), Ciudad de Mexico, México

**Keywords:** Diabetes mellitus, Continuous glucose monitoring, Ambulatory glucose profile, Digital ecosystem, Recommendations

## Abstract

**Purpose:**

This expert consensus provides a standardized methodology for interpreting continuous glucose monitoring (CGM) data to optimize diabetes management. It aims to help healthcare professionals recognize glycemic patterns and apply targeted interventions based on real-time glucose metrics.

**Methods:**

A systematic literature review informed expert panel discussions. Specialists from Latin America assessed CGM interpretation challenges, reviewed key metrics, and reached consensus through an anonymous voting process. The recommendations align with international guidelines while addressing regional limitations in technology access and healthcare infrastructure.

**Results:**

Reliable CGM data interpretation requires at least 70% sensor use over 14 days. The Ambulatory Glucose Profile (AGP) report serves as the primary tool, offering essential metrics such as time in range (TIR), time below range (TBR), time above range (TAR), coefficient of variation (CV), and glucose management indicator (GMI). Identifying hyperglycemia, hypoglycemia, and glucose variability allows for personalized treatment adjustments. The panel adopted international glycemic targets, adapting them to Latin American settings. The time in tight range (TITR) was considered but not included due to limited supporting evidence and regional barriers to advanced CGM technology.

**Conclusions:**

Standardized CGM interpretation improves glycemic control and treatment decisions. These recommendations provide a structured approach to diabetes care, aiming to enhance clinical outcomes and address healthcare disparities in Latin America.

**Supplementary Information:**

The online version contains supplementary material available at 10.1186/s13098-025-01702-y.

## Introduction

Diabetes mellitus is a leading cause of morbidity and mortality worldwide, with a significant impact in Latin America. Despite the availability of clinical practice guidelines in the region [1], no specific recommendations integrate objective glycemic metrics to guide therapeutic goals. Continuous glucose monitoring (CGM) has transformed diabetes care by providing real-time data on glucose fluctuations, offering a dynamic and individualized approach to treatment [2, 3]. However, CGM interpretation remains complex, particularly in Latin America, where healthcare infrastructure and access to technology vary widely.

To address this gap, a structured expert consensus was developed by a panel of diabetes specialists from Argentina, Brazil, Chile, Colombia, and Mexico, supported by an independent methodological team. The process was informed by a systematic literature review on CGM data interpretation, with a focus on AGP metrics. Experts initially worked independently and blinded to each other’s inputs, recording observations on common glycemic patterns and therapeutic responses. These preliminary insights were discussed in an in-person meeting, where final recommendations were collaboratively refined and subjected to anonymous voting using a five-point Likert scale. This structured approach ensured a rigorous and transparent process while minimizing bias.

The rationale for this consensus is reinforced by recent evidence supporting CGM-based glycemic assessment. Montaser et al. (2022) demonstrated that glycemic control can be effectively described using two principal dimensions: hyperglycemia exposure, measured through TAR and GMI, and hypoglycemia risk, assessed via TBR and CV [4]. More recently, Montaser et al. (2024) introduced CGM-derived dynamic markers such as entropy rate and Poincaré plot ellipse area, which may improve early detection of glycemic deterioration and diabetes progression [5]. These markers complement AGP metrics by incorporating temporal variability, potentially identifying dysglycemia before clinical diagnosis.

This expert consensus aims to provide clear, practical guidance for endocrinologists, diabetologists, family physicians, internists, general practitioners, and other healthcare professionals involved in diabetes management. The recommendations are designed to be applicable across different levels of the healthcare system, considering the regional challenges of Latin America. As the first regional consensus on CGM interpretation, this document seeks to standardize AGP-based decision-making to improve diabetes care and patient outcomes.

## Methods

This consensus was developed using a modified RAND-UCLA Appropriateness Method (RAM) [6]. Thus, an initial evidence base informed the process of independent participation among the panelists and a face-to-face session facilitated the discussion and formulation of consensus statements.

The evidence base for these recommendations was derived from a systematic literature review conducted in MEDLINE and Embase (July 2024) using MeSH terms related to diabetes, CGM, AGP, and glycemic targets. The review included clinical practice guidelines, systematic reviews, evidence-based recommendations, and observational studies published in English and Spanish, with no restrictions on publication date.

Two independent reviewers assessed studies based on predefined eligibility criteria, including quantitative data on CGM metrics, validated AGP interpretations, or clinical recommendations for diabetes management. Studies lacking original data, not focusing on CGM-based glycemic assessment, or presenting methodological limitations were excluded. Discrepancies were resolved by consensus.

To ensure relevance, the review prioritized recent publications, and additional targeted searches were conducted to address specific subtopics. The search strategy and the PRISMA evidence selection process are detailed in Supplementary Material 2.

The expert panel defined the scope of the consensus, identifying the target population and structuring the content into key thematic areas, including CGM data validity, interpretation metrics, individualized glycemic targets, glucose pattern recognition, and treatment decision-making in different clinical scenarios.

Experts first met virtually to review and validate the selected evidence, ensuring alignment with international guidelines such as the ATTD Consensus [7] and the ADA Standards of Care [8, 9]. The final recommendations remain consistent with the principles outlined in the updated 2025 ADA Standards [10]. These references were carefully considered to adapt CGM-based glycemic interpretation to the unique challenges of Latin America, addressing regional barriers in technology access, healthcare infrastructure, and clinical implementation.

Through a virtual collaborative document, each participant independently recorded observations on common glycemic patterns, underlying causes, and potential therapeutic interventions, while remaining blind to others' contributions. These insights were later discussed in an in-person meeting with representation from multiple Latin American countries. Final recommendations were refined through collaborative review and anonymously voted on using a 5-point Likert scale to minimize bias. Abbott provided logistical support but had no role in evidence selection, data analysis, or recommendation formulation. All participants declared potential conflicts of interest before contributing to the consensus.

## Results

**Fig. 1:**
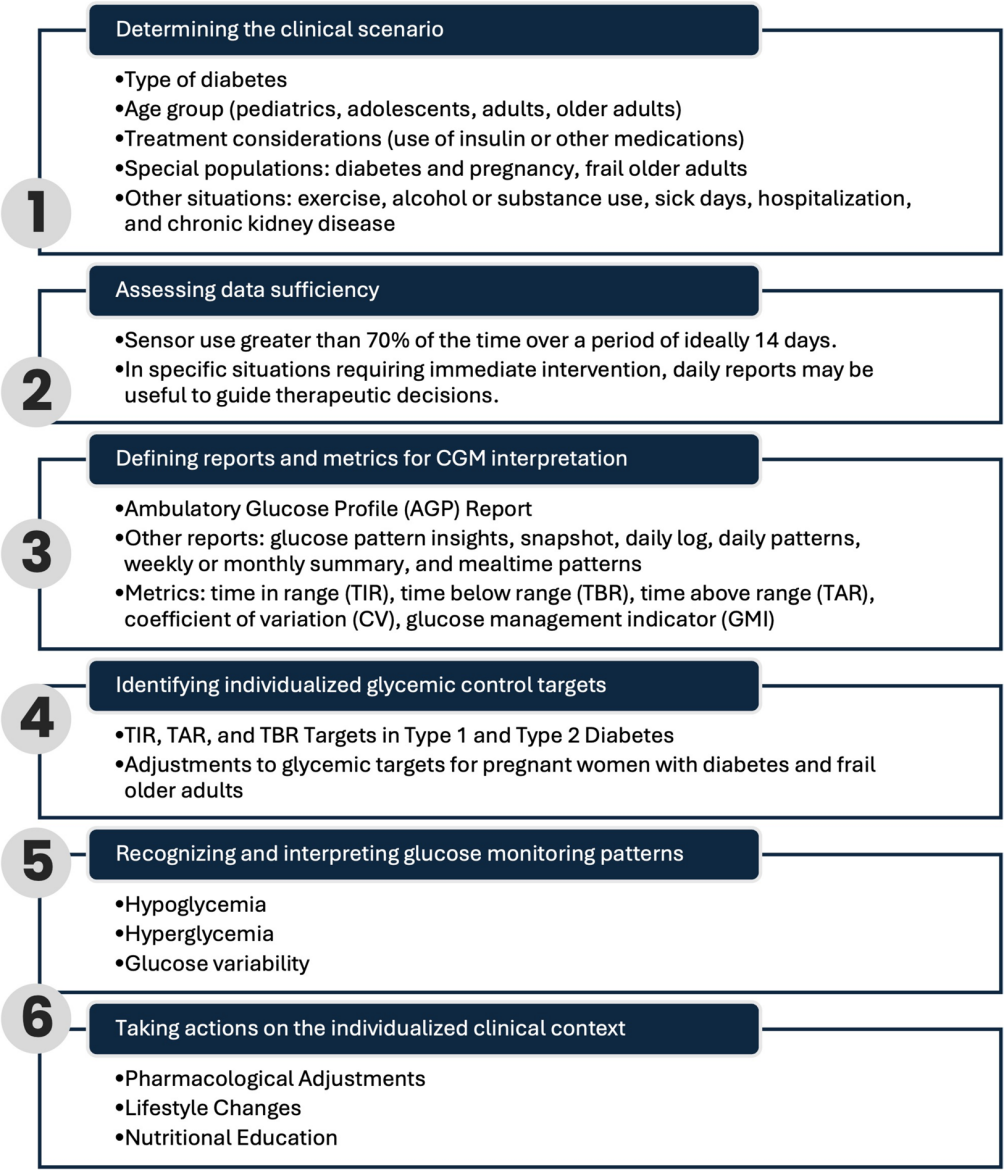
Steps for Interpreting Continuous Glucose Monitoring Data

The steps recommended for interpreting CGM data are presented in Fig. 1. Each aspect is discussed below, including the analysis and considerations provided by the expert panel, along with suggestions for action at each stage. The process, detailed in the following sections, consists of 1) determining the clinical scenario, 2) assessing the data sufficiency, 3) defining reports and metrics for CGM interpretation, 4) identifying individualized glycemic control targets, 5) recognizing and interpreting glucose monitoring patterns, and 6) taking actions on the individualized clinical context.

### Determining the clinical scenario

In line with the usual assessment approach for people with diabetes, the analysis of CGM reports should begin with an evaluation of the individual’s clinical profile, considering factors such as type of diabetes, age, and current treatment (e.g., oral antidiabetics, insulin, or non-insulin injectables). Additionally, special situations, such as gestational diabetes (GD), pregnancy in women with pre-existing diabetes, frailty, chronic kidney disease, and high risk of hypoglycemia, should be considered for an individualized interpretation of the CGM information. Other usually transient daily situations such as exercise, alcohol consumption, hospitalization, or sick days, should also be considered. While similar glycemic patterns (e.g., hypoglycemia, hyperglycemia, variability) may appear across these scenarios, the underlying causes and necessary interventions may differ, as discussed later.

### Assessing data sufficiency

**Fig. 2:**
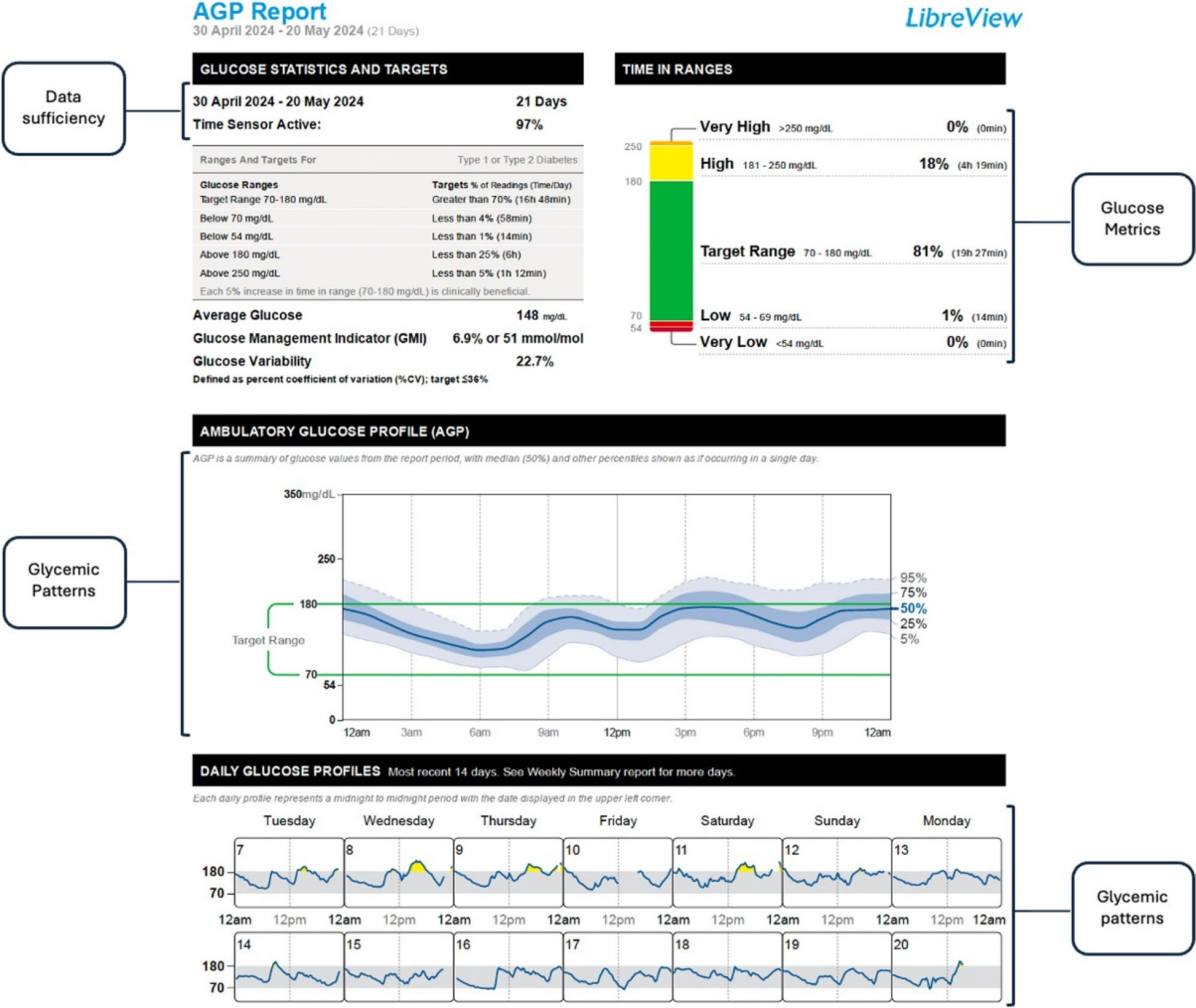
Key Metrics in the AGP Report for Continuous Glucose Monitoring. Adapted from Battelino T, Danne T, Bergenstal RM, et al. Clinical Targets for Continuous Glucose Monitoring Data Interpretation: Recommendations From the International Consensus on Time in Range. Diabetes Care. 2019;42(8):1593–1603

Once the clinical scenario has been defined, the next step in interpreting CGM data is to assess its validity and sufficiency. This information can be found in the header of the AGP report, as shown in Fig. 2. Adherence to sensor usage and proper utilization determines the reliability of the data obtained through monitoring. To ensure reliable glucose patterns, the sensor should be used more than 70% of the time, ideally over a 14-day period. However, in certain situations that require immediate intervention, such as hypoglycemia, clinical experts consider that daily reports may be sufficient to guide therapeutic decisions.

### Defining reports and metrics for CGM interpretation

CGM offers several metrics, as previously described, and this panel suggests a sequence for their detailed review and evaluation. The AGP Report provides a standardized summary of glucose patterns, making it a key tool for CGM analysis. Additional different reports in LibreView®, in recommended order of review, include Glucose Pattern Insights, Snapshot, Daily Log, Daily Patterns, Weekly Summary, Monthly Summary, and Mealtime Patterns [11]. Some cases may require alternative reports. For example, the Daily Log is particularly useful for hospitalized patients, those engaging in physical activity, or individuals at high risk of hypoglycemia. The selection of CGM reports should be based on clinical judgment and individual patient needs.

The key metrics for interpreting CGM data included in the AGP Report are TIR, TBR, TAR, CV, and GMI. The TIR is defined as glucose levels between 70 and 180 mg/dL, except for clinical scenarios related to gestation, as explained in Fig. 2. Current evidence has shown that CGM metrics, particularly TIR, TBR, and TAR, correlate with HbA1c, making them sensitive indicators of therapeutic efficacy and quality of the intervention [12]. Whereas, glucose variability, as reflected in CV, is closely associated with risk of hypoglycemia, and its analysis can help guide clinically relevant outcomes [13]. It is important to consider that variability may differ depending on the time frame analyzed and can be assessed throughout the day or during specific segments thereof. Similarly, the GMI serves as a reference for HbA1c values and supports the overall interpretation of the glycemic profile, facilitating the transition from traditional assessment methods to new CGM-based metrics [14]. Thus, the comprehensive and combined evaluation of GMI, average glucose, and the CV will collectively aid in more clearly determining the patient's glycemic profile. This triple target is the closest approach in conventional clinical practice to precision medicine.

### Identifying glycemic control targets

The panel decided to adopt the targets established by the International Consensus Report on Time in Range [7], widely used as a reference for diabetes management with CGM. This consensus [7] recommended glucose targets based on CGM data for different groups, such as people with type 1 diabetes (T1D) or type 2 diabetes (T2D), older or high-risk individuals with T1D or T2D, pregnant women with T1D, and women with GD or T2D during pregnancy. Of all the measures, TIR is particularly relevant because it has a high correlation with HbA1c, allows determining the individual metrics for each specific individual, and predicts the risk of complications in people with diabetes [15, 16]. TIR is also helpful in diabetes education because the concept is easier to understand than HbA1c. Glycemic targets for different clinical scenarios are based on the recommendations from the International Consensus on Time in Range [7].

Regarding high-risk groups for severe hypoglycemia, it is important to highlight that frail older adults—those with diminished physiological reserve and function—are particularly vulnerable to glucose fluctuations [17, 18]. In this population, the primary goal is to minimize hypoglycemia (< 1% of the time < 70 mg/dL) and prevent symptomatic hyperglycemia, prioritizing a TIR ≥ 50% within 70–180 mg/dL while maintaining glycemic variability within safe limits (CV ≤ 36%). Further research is needed to determine whether adjustments to current thresholds are warranted in specific clinical contexts.

The time in tight range (TITR), defined as glucose levels between 70 and 140 mg/dL, is considered a meaningful metric due to its strong correlation with TIR and its inverse association with the development of microvascular complications and cerebrovascular events in people with T1D [19, 20]. The panel considered including TITR, particularly for specific populations such as young children or those using automated insulin delivery. However, due to the limited supporting evidence, regional barriers to advanced CGM technology, and the lack of demonstrated clinical advantage over TIR, it was not included in the glycemic targets at this time.

For pregnant women with type 1 diabetes, the target is to maintain glucose levels between 63–140 mg/dL for at least 70% of the time, as recommended by the international consensus [7]. While some expert opinions suggest a TIR > 90% for pregnant women with type 2 diabetes or gestational diabetes, supporting evidence remains limited. Observational studies indicate that higher TIR correlates with improved perinatal outcomes [21, 22]. Given the variability in glucose regulation and the potential risk of hypoglycemia, glycemic targets should be individualized based on factors such as glycemic variability, average glucose, and overall maternal risk.

### Recognizing and interpreting glucose monitoring patterns

**Fig. 3:**
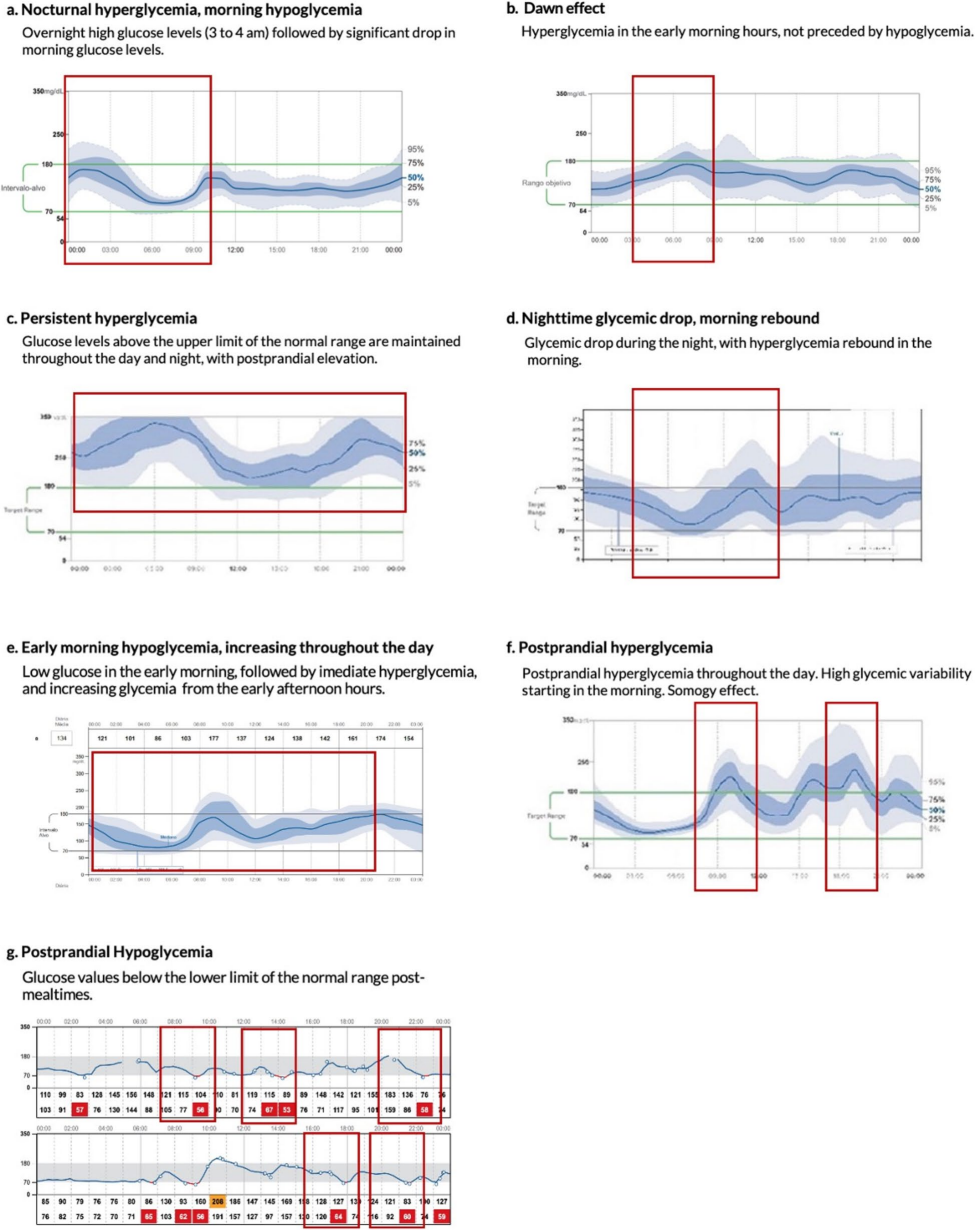
Common Glycemic Patterns in AGP Reports of People with Diabetes Mellitus

The glycemic patterns identified in the AGP Report are based on three easily recognizable trends: hypoglycemia, hyperglycemia, and glucose variability and the combination of these in a single record. In cases where more than one metabolic scenario exists, it is essential to identify hypoglycemia in the first place. Those glycemic patterns may occur across various clinical scenarios in people with diabetes, leading to different therapeutic approaches based on the underlying cause. In the AGP graph, which shows glycemic levels on a 24-h scale, it is essential to identify levels outside the target range and when these glycemic excursions occur (daytime baseline, nighttime baseline, prandial) for accurate interpretation. Examples of the most relevant glycemic patterns in the AGP Reports of people with diabetes are presented in Fig. 3.

Hyperglycemia is defined as glucose levels above the upper limit of the TIR and may occur at certain times of the day or night or in association with food intake. This pattern is often the result of suboptimal antidiabetic therapy, inappropriate insulin timing, problems related to carbohydrates intake (e.g., overconsumption, incorrect counting, or glycemic index), or stressful situations. Clinical conditions such as fever, gastroparesis, lipodystrophy, chronic kidney disease, and glucocorticoid use can also lead to hyperglycemic events and must be identified to achieve optimal glycemic goals [23–25].

Hypoglycemia, on the other hand, is defined as blood glucose levels below the lower limit of the TIR and may also occur at certain times of the day or night or in relation to food intake omission or alcohol consumption. This glycemic pattern is usually due to overdose or overlapping of insulin, overuse of antidiabetic drugs, aerobic physical activity, or incorrect carbohydrate counting. It is important to accurately identify the frequency of the event (pattern over time of the record), the timing (time of day), the depth, the duration (severities determined by levels) and circumstances of hypoglycemic events to identify the causes and make the necessary pharmacologic or dietary adjustments [26].

Continuous data of a dynamic variable over time allows us to obtain the standard deviation (SD) and interquartile ranges (IQR). Thus, mixed or variable patterns are characterized by a CV greater than 36% in AGP metrics. High glucose variability in people with diabetes is common in transient situations such as exercise, alcohol intake, hospitalization or may occur with irregular adherence to pharmacological or nutritional therapy. These cases usually present high variability with delayed hypoglycemia (4–6 h postprandial), requiring different management approaches. Analysis should begin with daily CGM recording, focusing on the 5–95% and 25–75% ranges, along with event tagging, to support glucose interpretation and guide individualized therapy.

### Taking actions on the individualized clinical context

Once glycemic patterns have been identified, it is important to explore their causes. As mentioned earlier, these scenarios are not mutually exclusive, and in most cases, more than one can occur in the same person. Problems with a pattern should always be evaluated; that is, those that repeat over time. It is always recommended to assess the segment prior to the clinically significant event, ideally 4 to 6 h before. A glycemic pattern or its combination may be related to medication regimens, medication adherence, behaviors, or the disease itself. These factors may be present in the basal or prandial segment of the record. The causes and corrective actions are variable and decisions regarding pharmacologic or dietary adjustments, lifestyle changes, education, and other interventions must be made according to the individual clinical circumstances. Tables 1, 2, and 3 show some of the most common glycemic patterns on CGM in people with T1D, T2D, and gestational diabetes, along with the identifiable causes in each clinical scenario and the corresponding therapeutic interventions. The group of experts agreed that identifying and interpreting these patterns in the AGP graph is a useful tool for decision-making in therapeutic interventions. They emphasize that a major obstacle with the interpretation of records is capturing all the exposure factors that generate changes in glucose, such as meals, exercise timing, medications taken or applied like insulin and their timing with meals, corrections, etc. It would be essential to have automation in the recording of these exposure factors.

**Table 1:** Identification of Glycemic Patterns, Causes, and Interventions in Patients with Type 1 Diabetes Using Continuous Glucose Monitoring

Glycemic pattern	Description	Possible Causes	Suggested Interventions
	Hyperglycemia postprandial, glycemic drop during the night	• Inadequate basal insulin dose	• Review and adjust evening basal insulin doses
		• Inadequate ICR or CH count at dinner	• Insulin dose adjustment based on CH count
	Hyperglycemia in the early morning hours, not preceded by hypoglycemia, in people with T1D	Dawn phenomenon	• Basal insulin dose adjustment • Correction bolus in early morning
	Persistent hyperglycemia in adolescents or young adults with T1D	• Missed/insufficient dose of prandial bolus	• Education on pre-meal insulin administration • Cognitive behavioral intervention
		• Missed or underdosed basal insulin	• Education on basal insulin administration • Cognitive behavioral intervention
	Postprandial hyperglycemia throughout the day. High glycemic variability starting in the morning, in T1D	• Inadequate ICR or CH count	• Education on CH count or adjust ICR
		• Inadequate bolus timing	• Administer pre-prandial bolus in advance
		• High intake of CH or CH with a high glycemic index	• Education on nutrition • Cognitive behavioral intervention
		• High protein/fat foods	• Split bolus administration
		• Bolus skip	• Education on bolus administration
	Postprandial hypoglycemia in people with T1D	• Inaccurate CH count, not eating as expected	• Education on CH count or adjust ICR • Instruct the patient to administer insulin only for food they are sure they will eat
		• Physical activity	• Reduce insulin dose (10–50%)
		• Insulin overdose	• Instruct the patient to administer insulin only for the food they are certain they will eat
		• Inadequate prandial bolus timing	• Education on pre-meal insulin administration • Cognitive behavioral intervention
		• Very low insulin sensitivity factor	• Increase the insulin sensitivity factor to decrease dose
		• Application of a second bolus during the active life of insulin (*Stacking*)	• Education on insulin administration
		• Associated clinical conditions (gastroparesis, lipodystrophy, CKD, among others)	• Individualized diagnosis and management
	Nighttime glycemic drop, morning rebound hyperglycemia in people with T1D	• Excessive basal insulin • Somogyi effect	• Basal insulin reduction

T1D = Type 1 Diabetes Mellitus; CH = Carbohydrates; CKD = Chronic Kidney Disease; ICR = Insulin-To-Carbohydrate Ratio

**Table 2:** Identification of Glycemic Patterns, Causes, and Interventions in Patients with Type 2 Diabetes Using Continuous Glucose Monitoring

Glycemic pattern	Description	Causes	Interventions
	Nighttime glycemic drop, morning rebound in adults with T2D on insulin therapy	• Excessive basal insulin	• Basal insulin reduction
		• Low protein and high carbohydrate load in meals, especially in the morning	• Reduce CH intake at breakfast • Consider protein supplementation if necessary
	Persistent hyperglycemia through the day in T2D adults without insulin therapy	• Suboptimal antidiabetic therapy	• Optimize treatment, add other agents or basal insulin
		• Inadequate endogenous insulin response to CH loads	• Reduce the amount of CH in meals • Lifestyle changes
		• Difficulty reducing CH intake	• Education on nutrition • Intensify treatment with antidiabetic drugs with prandial impact
		• High CH intake	• Education on nutrition and lifestyle changes
	Postprandial hyperglycemia, in T2D adults without insulin	• Effect of atypical antipsychotics or glucocorticoids	• Anti-diabetic with prandial effect
		• Suboptimal antidiabetic therapy	• Optimize treatment
		• Lack of adherence to pharmacological and nutritional therapy	• Intensify the treatment with antidiabetic drugs with prandial impact • Education on nutrition and lifestyle changes

T2D = type 2 diabetes mellitus; CH = carbohydrates; GLP1 = glucagon-like peptide type 1

**Table 3:** Identification of Glycemic Patterns, Causes, and Interventions in Patients with Diabetes and Pregnancy Using Continuous Glucose Monitoring

Glycemic pattern		Causes	Interventions
	Hypoglycemia in women with T1D during the first trimester of pregnancy	• Reduced carbohydrate intake associated with emesis gravidarum	• Avoid prolonged fasting • Treat emesis with diet and medication • Decrease insulin dose
		• Increased sensitivity to insulin in the first trimester (in those on insulin treatment)	• Decrease basal insulin dose • Increase ICR and insulin sensitivity factor
	Postprandial hyperglycemia in women with T1D during the third trimester of pregnancy or in women with gestational diabetes	• High intake of CH with high glycemic index or mismatch between insulin dose and CH intake	• Education on reducing carbohydrate load and glycemic index • Ensure adequate intake of proteins and healthy fats
		• Need to use or adjust the dose of rapid-acting insulin	• Adjust ICR and insulin sensitivity factor • Verify proper timing of bolus administration • Initiate rapid-acting insulin approved for pregnancy • Encourage exercise whenever possible
		• Use of glucocorticoids for lung maturation	• Adjust treatment as needed
	Sustained hyperglycemia in women with T2D during pregnancy	• Discontinuation antidiabetic medications due to contraindication for use during pregnancy	• Initiate insulin early
		• Progressive increase in insulin resistance	• Initiate insulin early • Prompt adjustment of basal insulin dose and prandial bolus as needed • Encourage exercise whenever possible
		• Inadequate diet	• Education on carbohydrate intake and glycemic index

T1D = Type 1 diabetes mellitus; T2D = type 2 diabetes mellitus; CH = carbohydrates; ICR = Insulin-To-Carbohydrate Ratio

In terms of diet, therapeutic adjustments should address carbohydrate counting, to avoid mismatch between insulin dose, carbohydrate intake and the identification of high glycemic index carbohydrates. Improving glycemic control is also an essential component of interventions to reduce the risk of cardiovascular events and other complications related to diabetes [27, 28].

In individuals receiving multiple doses of insulin, it is necessary to consider the timing of prandial insulin administration, the insulin/carbohydrate ratio, the insulin sensitivity factor. In older people, therapeutic adjustments require special attention due to factors such as variable intake, polypharmacy, sarcopenia and decreased muscle and liver glycogen stores, which increase their sensitivity to insulin [29]. Clinicians or patients using the device in real-time can use trend arrows or CGM alarms for greater precision in therapeutic adjustments. This is not a substitute for the usual bolus calculations, but they will facilitate corrective actions in the treatment itself (e.g., reducing or increasing insulin boluses) or behavioral actions (e.g., consuming carbohydrates, exercise, bolus timing, among others) in different clinical scenarios throughout the day (pre-prandial scenario and between meals or correction scenarios).

Pregnancy-related diabetes may show glycemic patterns like those seen in T1D or T2D, including prandial hyperglycemia or hypoglycemia. However, pregnancy-related changes in glycemic targets must be considered when evaluating the causes. Postprandial hyperglycemia, especially in the early morning hours, is common in gestational diabetes [30, 31], and the use of glucocorticoids for fetal lung maturation can increase post-prandial glucose levels, leading to rapid changes in insulin requirements [32, 33]. Treatment adjustments should prioritize dietary management, particularly glycemic index control, along with optimizing rapid-acting insulin dosing and increasing physical activity [34].

In special situations caused by temporary circumstances, interventions depend on the type of stressor involved. People who exercise have increased insulin sensitivity during and after exercise [35, 36]. In addition, lack of carbohydrate intake can lead to hypoglycemia, and interventions should be tailored to the type, intensity, and duration of exercise. Interventions include insulin adjustments, increased monitoring during and after exercise, and preventive carbohydrate ingestion. On the other hand, glucose variability associated with the consumption of alcohol and other substances should be managed with intensified monitoring and preventive corrective actions.

For ill individuals, it is important to interpret CGM data, fundamentally the daily report, in the context of hemodynamic instability, ICU stay, continuous enteral nutrition, pain, fever, post-operative conditions, among others. In these cases, insulin schedules should be adjusted, considering the acute event, the administration of concomitant medications, and the possibility of cyclical situations, using trend arrows to guide adjustments.

## Conclusions

Effective use of CGM in diabetes care depends on accurate data interpretation to guide treatment decisions. This process starts with assessing the individual’s clinical profile, including diabetes type, age, treatment, and temporary conditions that may affect glucose patterns. Reliable data require a minimum of 70% sensor use over 14 days, though in high-risk situations, such as frequent hypoglycemia, five days may be enough for immediate adjustments.

The AGP Report provides a standardized summary of glucose patterns. Key metrics, TIR, TBR, TAR, CV, and GMI, help assess glycemic control and treatment response. Standard targets aim to keep glucose between 70 and 180 mg/dL, with specific adjustments for pregnancy and older adults at risk of hypoglycemia.

Latin America has wide disparities in healthcare access, technology availability, and economic resources. While CGM adoption is increasing, cost and infrastructure remain barriers, especially in low-resource settings. Expanding access will require policies that support reimbursement, provider training, and patient education. Integrating CGM into diabetes care at all levels, including primary care, can help improve outcomes and reduce complications, making these recommendations relevant beyond specialized centers. In addition, as CGM is likely to become more widely used, expert recommendations will be even more important to ensure its successful integration and maximise its benefits in different healthcare settings.

Recognizing and interpreting patterns of hypoglycemia, hyperglycemia, and glucose variability allows for timely adjustments that help maintain stable glucose levels. Personalized interventions based on these insights can improve long-term outcomes and quality of life for people with diabetes.

## Supplementary Information

Additional file1: Appendix A: Comprehensive Glucose Data Analysis with LibreView.

Additional file 2: Appendix B: search strategy and the PRISMA evidence selection process.

## Data Availability

No datasets were generated or analysed during the current study.

## Abbreviations

AGP: Ambulatory Glucose Profile
CGM: Continuous glucose monitoring
CH: Carbohydrates
CKD: Chronic Kidney Disease
CV: Coefficient of Variation
FDA: U.S. Food and Drug Administration
GD: Gestational diabetes
GLP1: Glucagon-like peptide type 1
GMI: Glucose Management Indicator
GPI: Glucose Pattern Insights
HbA1c: Glycated hemoglobin
ICGM: Integrated continuous glucose monitor
ICR: Insulin-To-Carbohydrate Ratio
ICU: Intensive care unit
IQR: Interquartile ranges
T1D: Type 1 diabetes
T2D: Type 2 diabetes
TAR: Time above range
TBR: Time below range
TITR: Time in tight range
TIR: Time in range
