# Supplementary material for: How to take action beyond ambulatory glucose profile: Latin American expert recommendations on CGM data interpretation
Source: Diabetol Metab Syndr. 2025 May 8;17:149. doi: 10.1186/s13098-025-01702-y (PMC12060294; doi:10.1186/s13098-025-01702-y)
Supplement: Supplementary file 1 — Additional file1: Appendix A: Comprehensive Glucose Data Analysis with LibreView. [file 13098_2025_1702_MOESM1_ESM.docx]

# **Appendix A. Comprehensive Glucose Data Analysis with LibreView**

| **AGP Report [ENG]**  **Informe del AGP [ESP]**  **Relatório do AGP [POR]** | **Glucose Pattern Insights [ENG]**  **Visualización del patrón de glucosa [ESP]**  **Perspectivas do Padrão de Glicose [POR]** | **Snapshot [ENG]**  **Instantánea [ESP]**  **Panorama [POR]** | **Daily Log [ENG]**  **Registro diario [ESP]**  **Registo diário [POR]** |
| --- | --- | --- | --- |
| 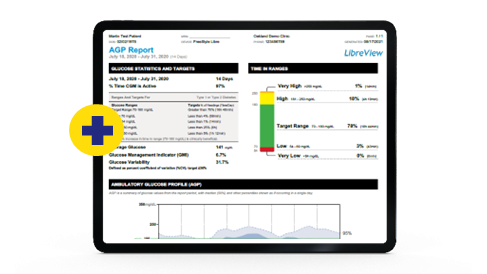 | 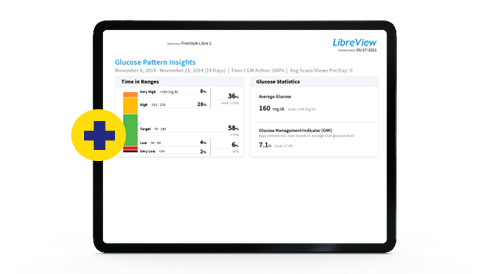 | 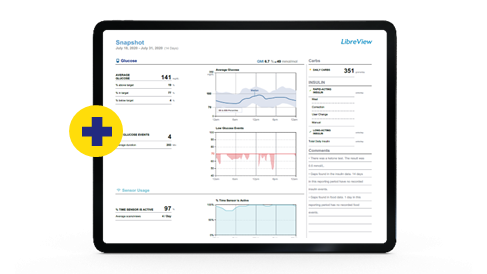 | 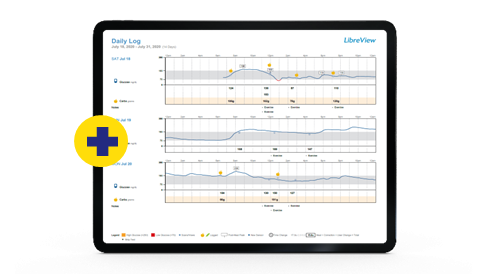 |
| The AGP Report is a standardized report developed by the International Diabetes Center (IDC) and shows a standard set of information and graphs.  It includes:   - Glucose Statistics and Targets - Glucose Management Indicator (GMI) - Time in Ranges - Ambulatory Glucose Profile (AGP) - Daily Glucose Profiles | The Glucose Pattern Insights report offers an interpretation of the AGP  by highlighting glycemic patterns and supplying medication and lifestyle considerations to address them.  It includes:   - Glucose Statistics - Time in Ranges - Considerations for the Clinician (highest priority glycemic pattern, medication and Lifestyle considerations) - Glucose Patterns | The Snapshot report shows a summary of glucose, sensor usage, carbohydrate, insulin, and quantitative hypoglycemic data for the 14-day time period.  It includes:   - Average glucose - Sensor usage (%) - Daily Carbs - Insulin - Comments (patterns in the data) - Low Glucose Events | The Daily Log report shows detailed glucose, carbohydrate, insulin and other data that a patient has logged and when the data were logged for each day in the 14-day period. This report is the only report in this set that includes strip test results.  It includes:   - One day of data - Glucose values - Scans/Views - Individual readings - Rapid-acting insulin - Long-acting insulin |

| **Daily Patterns [ENG]**  **Patrones diarios [ESP]**  **Padrões diários [POR]** | **Weekly Summary [ENG]**  **Resumen semanal [ESP]**  **Resumo semanal [POR]** | **Monthly Summary [ENG]**  **Resumen mensual [ESP]**  **Resumo mensal [POR]** | **Mealtime Patterns [ENG]**  **Patrones de comida [ESP]**  **Padrões de refeição [POR]** |
| --- | --- | --- | --- |
| 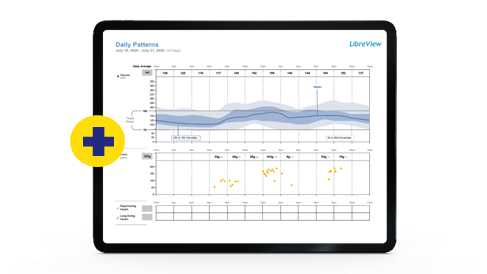 | 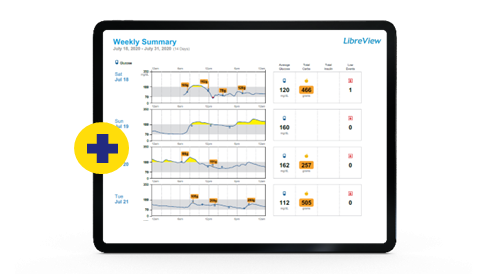 | 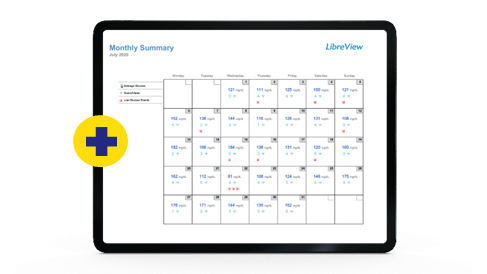 | 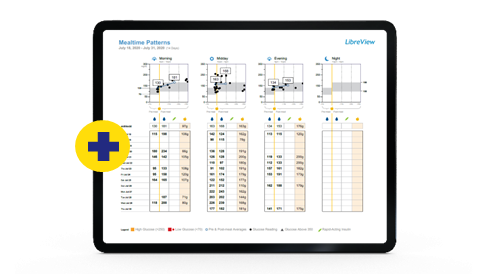 |
| The Daily Patterns report shows glucose, carbohydrate and insulin data over the “typical” day based on all days within the 14-day period. It includes the AGP, a graph of the 5th, 25th, 50th (median), 75th and 95th percentiles of glucose readings.  It includes:   - Average glucose * - Carbohydrates * - Insulin *   *Data average from all days in the reporting period selected as well as the average for every two hours of the day over a 24-hour period. | The Weekly Summary report shows daily glucose, insulin and carbohydrates data shown in a weekly format. This report is very similar to the Daily Log report, except that it summarizes a full week's worth of data per page.  It includes:   - One graph per day - Glucose trend - Total Carbohydrates - Total Insulin - Low Glucose Events - Scans/Views | The Monthly Summary report shows average glucose, number of low glucose events and sensor usage data in a calendar format for each month. Sensor usage data includes a total number of scans or views per day. | Shows glucose, carbohydrate, and insulin data for “typical” meals, based on all meal tags within the 14-day period. Reveals patterns for patients who log rapid-acting insulin and meal tags on their device.  It includes:   - Time blocks (data for different times of day. Each time block represents one-hour premeal and three hours post-meal) - Insulin averages - Calculator settings - Pre- & post-prandial glucose   *It is necessary to add information (meals, insulin, carbohydrates) in the notes to visualize the data. |
