# Supplementary material for: How to take action beyond ambulatory glucose profile: Latin American expert recommendations on CGM data interpretation
Source: Diabetol Metab Syndr. 2025 May 8;17:149. doi: 10.1186/s13098-025-01702-y (PMC12060294; doi:10.1186/s13098-025-01702-y)
Supplement: Supplementary file 2 — Additional file 2: Appendix B: search strategy and the PRISMA evidence selection process. [file 13098_2025_1702_MOESM2_ESM.docx]

**Appendix B: search strategy and the PRISMA evidence selection process**

**Search strategies in electronic databases**

**Database: Medline on PubMed**

**Search date: August 2024**

**Results:**

1 "diabete"[All Fields] OR "diabetes mellitus"[MeSH Terms] OR ("diabetes"[All Fields] AND "mellitus"[All Fields]) OR "diabetes mellitus"[All Fields] OR "diabetes"[All Fields] OR "diabetes insipidus"[MeSH Terms] OR ("diabetes"[All Fields] AND "insipidus"[All Fields]) OR "diabetes insipidus"[All Fields] OR "diabetic"[All Fields] OR "diabetics"[All Fields] OR "diabets"[All Fields] 1,000,302

2 ("flash"[All Fields] OR "flash s"[All Fields] OR "flashed"[All Fields] OR "flashes"[All Fields] OR "flashing"[All Fields] OR "flashings"[All Fields]) AND ("glucose"[MeSH Terms] OR "glucose"[All Fields] OR "glucoses"[All Fields] OR "glucose s"[All Fields]) AND ("monitor"[All Fields] OR "monitor s"[All Fields] OR "monitorable"[All Fields] OR "monitored"[All Fields] OR "monitoring"[All Fields] OR "monitoring s"[All Fields] OR "monitorings"[All Fields] OR "monitorization"[All Fields] OR "monitorize"[All Fields] OR "monitorized"[All Fields] OR "monitors"[All Fields]) 756

3 "FGMS"[All Fields] 203

4 "continuous glucose monitoring"[MeSH Terms] OR ("continuous"[All Fields] AND "glucose"[All Fields] AND "monitoring"[All Fields]) OR "continuous glucose monitoring"[All Fields] 9,971

5 "CGM"[All Fields] 5,461

6 "agp"[All Fields] 5,047

7 ("ambulatories"[All Fields] OR "ambulatory"[All Fields]) AND ("glucose"[MeSH Terms] OR "glucose"[All Fields] OR "glucoses"[All Fields] OR "glucose s"[All Fields]) AND ("profile"[All Fields] OR "profiled"[All Fields] OR "profiler"[All Fields] OR "profilers"[All Fields] OR "profiles"[All Fields] OR "profiling"[All Fields] OR "profilings"[All Fields]) 657

8 "interpret"[All Fields] OR "interpretability"[All Fields] OR "interpretable"[All Fields] OR "interpretating"[All Fields] OR "interpretation"[All Fields] OR "interpretation s"[All Fields] OR "interpretational"[All Fields] OR "interpretations"[All Fields] OR "interpretative"[All Fields] OR "interpreted"[All Fields] OR "interpreter"[All Fields] OR "interpreter s"[All Fields] OR "interpreters"[All Fields] OR "interpreting"[All Fields] OR "interpretive"[All Fields] OR "interpretively"[All Fields] OR "interprets"[All Fields] 738,494

9 "data basel"[Journal] OR "brown univ dig addict theory appl"[Journal] OR "data"[All Fields] 6,605,797

10 "guide"[All Fields] OR "guided"[All Fields] OR "guides"[All Fields] OR "guiding"[All Fields] 576,224

11 "reportable"[All Fields] OR "reporting"[All Fields] OR "reportings"[All Fields] OR "research report"[MeSH Terms] OR ("research"[All Fields] AND "report"[All Fields]) OR "research report"[All Fields] OR "report"[All Fields] OR "reported"[All Fields] OR "reports"[All Fields] 6,978,574

12 Search #2 OR #3 OR #4 OR #5 OR #6 OR #7 17,555

13 Search #8 OR #9 OR #10 OR #11 12,705,874

14 Search #1 AND #12 AND #13 5,249

15 Search #14 AND (y_10[Filter]) 285

**Database: LILACS on**

**Search date: August 2024**

**Results:**

1 diabetes mellitus AND instance:"regional" 637.778

2 (continuous glucose monitoring) OR (cgm) OR (fgms) OR (flash monitor*) OR (agp) OR (ambulatory glucose profile) AND instance:"regional" 22.790

3 (guide) OR (report*) OR (data interpretation) AND instance:"regional" 11.215.615

4 (diabetes mellitus) AND ((continuous glucose monitoring) OR (cgm) OR (fgms) OR (flash monitor*) OR (agp) OR ((ambulatory glucose profile))) AND instance:"regional" 8.218

5 (diabetes mellitus) AND ((continuous glucose monitoring) OR (cgm) OR (fgms) OR (flash monitor*) OR (agp) OR ((ambulatory glucose profile))) AND ((guide) OR (report*) OR (data interpretation)) AND instance:"regional"  2.866

6 Search #5 NOT MEDLINE 83

**PRISMA 2020 flow diagram**

Records removed *before screening*:

Duplicate records removed (n = 25)

Records identified from:

Databases (n = 368)

**Identification**

Records screened

(n = 343)

Records excluded

(n = 264)

Reports sought for retrieval

(n = 79)

Reports not retrieved

(n = 0)

**Screening**

Reports excluded: 56

No intervention: 6

No specific outcomes:19

No additional information: 31

Reports assessed for eligibility

(n = 79)

Documents included in review

(n =23)

**Included**

**Selected references**

Al-Gadi, I., Menon, S., Lyons, S. K., & DeSalvo, D. J. (2021). Beyond A1C: a practical approach to interpreting and optimizing continuous glucose data in youth. Diabetes Spectrum, 34(2), 139-148.

Aleppo, G., & Webb, K. (2019). Continuous glucose monitoring integration in clinical practice: a stepped guide to data review and interpretation. *Journal of diabetes science and technology*, *13*(4), 664-673.

Battelino, T., Danne, T., Bergenstal, R. M., Amiel, S. A., Beck, R., Biester, T., ... & Phillip, M. (2019). Clinical targets for continuous glucose monitoring data interpretation: recommendations from the international consensus on time in range. *Diabetes care*, *42*(8), 1593-1603.

Bergenstal, R. M., Ahmann, A. J., Bailey, T., Beck, R. W., Bissen, J., Buckingham, B., ... & Wesley, D. M. (2013). Recommendations for standardizing glucose reporting and analysis to optimize clinical decision making in diabetes: the Ambulatory Glucose Profile (AGP).

Richard M. Bergenstal; Understanding Continuous Glucose Monitoring Data. ADA Clinical Compendia 1 August 2018; 2018 (1): 20–23.

Bergenstal, R. M., Simonson, G. D., & Heinemann, L. (2022). More green, less red: how color standardization may facilitate effective use of CGM data. *Journal of Diabetes Science and Technology*, *16*(1), 3-6.

Borot, S., Benhamou, P. Y., Atlan, C., Bismuth, E., Bonnemaison, E., Catargi, B., ... & Hanaire, H. (2018). Practical implementation, education and interpretation guidelines for continuous glucose monitoring: a French position statement. *Diabetes & metabolism*, *44*(1), 61-72.

Brown, S. A., Basu, A., & Kovatchev, B. P. (2019). Beyond HbA1c: using continuous glucose monitoring metrics to enhance interpretation of treatment effect and improve clinical decision‐making. *Diabetic Medicine*, *36*(6), 679-687.

Carlson, A. L., Mullen, D. M., & Bergenstal, R. M. (2017). Clinical use of continuous glucose monitoring in adults with type 2 diabetes. *Diabetes technology & therapeutics*, *19*(S2), S-4.

Cetina-Canto, J. A., Yépez-Rodríguez, A. E., Barrientos-Pérez, M., Márquez-Rodríguez, E., Navarro-Lara, A., Escalante-Pulido, M., ... & FLORES-CALOCA, Ó. S. C. A. R. (2022). Guía y recomendaciones para el uso del sistema flash de monitoreo continuo de glucosa (iMCG). *Revista Mexicana de Endocrinología, Metabolismo y Nutrición*, *9*.

Chico, A., Aguilera, E., Ampudia-Blasco, F. J., Bellido, V., Cardona-Hernández, R., Escalada, F. J., ... & González-Blanco, C. (2020). Clinical approach to flash glucose monitoring: an expert recommendation. *Journal of diabetes science and technology*, *14*(1), 155-164.

Coronel-Restrepo, N., Ramirez-Rincón, A., Palacio, A., Delgado, M. R., & Botero, J. F. (2023). Interpretación del monitoreo continuo de glucosa: una visión práctica. *Revista Colombiana de Endocrinología, Diabetes & Metabolismo*, *10*(1).

Czupryniak, L., Dzida, G., Fichna, P., Jarosz-Chobot, P., Gumprecht, J., Klupa, T., ... & Zozulinska-Ziolkiewicz, D. A. (2022). Ambulatory glucose profile (AGP) report in daily care of patients with diabetes: practical tips and recommendations. *Diabetes Therapy*, *13*(4), 811-821.

Dagdelen, S., Deyneli, O., Dinccag, N., Ilkova, H., Osar Siva, Z., Yetkin, I., & Yilmaz, T. (2022). Expert panel recommendations for use of standardized glucose reporting system based on standardized glucometrics plus visual ambulatory glucose profile (AGP) data in clinical practice. *Frontiers in Endocrinology*, *12*, 663222.

Doupis, J., & Horton, E. S. (2022). Utilizing the new glucometrics: a practical guide to ambulatory glucose profile interpretation. *touchREVIEWS in Endocrinology*, *18*(1), 20.

Edelman, S. V., Cavaiola, T. S., Boeder, S., & Pettus, J. (2021). Utilizing continuous glucose monitoring in primary care practice: what the numbers mean. *Primary Care Diabetes*, *15*(2), 199-207.

Ekhlaspour, L., Tabatabai, I., & Buckingham, B. (2019). A review of continuous glucose monitoring data interpretation in the age of automated insulin delivery. *Journal of Diabetes Science and Technology*, *13*(4), 645-663.

Gibb, F. W., Jennings, P., Leelarathna, L., & Wilmot, E. G. (2020). AGP in daily clinical practice: a guide for use with the FreeStyle Libre flash glucose monitoring system. *British Journal of Diabetes*, *20*(1), 32-40.

Hammond, P. (2016). Interpreting the ambulatory glucose profile. *British Journal of Diabetes*, *16*, S10-S15.

Kröger, J., Reichel, A., Siegmund, T., & Ziegler, R. (2020). Clinical recommendations for the use of the ambulatory glucose profile in diabetes care. *Journal of Diabetes Science and Technology*, *14*(3), 586-594.

Kruger, D. F., Edelman, S. V., Hinnen, D. A., & Parkin, C. G. (2019). Reference guide for integrating continuous glucose monitoring into clinical practice. *The Diabetes Educator*, *45*(1_suppl), 3S-20S.

Lin, R., Brown, F., & Ekinci, E. I. (2022). The ambulatory glucose profile and its interpretation. *The Medical Journal of Australia*, *217*(6), 295.

Szmuilowicz, E. D., & Aleppo, G. (2022). Stepwise approach to continuous glucose monitoring interpretation for internists and family physicians. *Postgraduate medicine*, *134*(8), 743-751.
